# Effect of dietary *Moringa oleifera *on production performance and gut health in broilers

**DOI:** 10.5455/javar.2024.k782

**Published:** 2024-06-08

**Authors:** Shumaila Gul, Fida Hussain, Raheela Taj, Asad Ullah

**Affiliations:** 1 Department of Chemical and Life Sciences, Qurtuba University of Science and Information Technology, Peshawar, Pakistan; 2 Institute of Chemical Sciences, University of Peshawar, Peshawar, Pakistan; 3 College of Veterinary Sciences and Animal Husbandry, Abdul Wali Khan University, Mardan, Pakistan

**Keywords:** Birds, goblet cell, health, medicinal plants, prebiotic

## Abstract

**Objective:**

In the present research work, we examined the dietary *Moringa oleifera *effect on gut health and growth traits in chickens.

**Materials and Methods:**

There were 280 chicks (day old) that were weighted and allotted uniformly in seven groupings, each containing eight replicates (*n =* 5). Birds were supplemented with *M. oleifera *leaf extract (MLE) and seed extract (MSE) for 35 days. Group I was the control (fed merely basal diets), while Group II received 0.8% MLE, Group III was given 0.8% MSE, Group IV was given 1.2% MLE, Group V was given 1.2% MSE, Group VI was given 0.8% MLE + 0.8% MSE, and Group VII was given 1.2% MLE + 1.2% MSE. At the end of the fifth week, two chickens were selected from each replica, and samples (small intestine and ileal ingesta) were collected.

**Results:**

The chicken diet with MLE and MSE supplements saw significant improvement (*p* < 0.05) in both feed conversion ratio (FCR) and body weight gain (BWG). In the small intestine (duodenal, jejunal, and ileal), dietary MLE and MSE supplements significantly increased (*p* < 0.05) the surface area of the villus and the ratio of their height/crypt depth in comparison to the control group. The MLE and MSE supplements significantly increased (*p* < 0.05) the total goblet cell counts in the small intestine. The Lactobacillus* spp. *count was significantly improved (*p* < 0.05) and reduced (*p* < 0.05) in *Escherichia coli* counts when the bird diet was supplemented with MLE (0.8%) and MSE (0.8%).

**Conclusion:**

Results indicated that *M. oleifera* leaf and seed extract diet improved the growth trait and gut health in chickens.

## Introduction

Long-term health advantages are increased by therapeutic plants that contain a variety of phytochemicals and bioactive substances such as vitamins, lipids, alkaloids, polyphenols, trace metal ions, carotenoids, carbs, and proteins [1]. When describing botanicals, essential oils, and extracts produced from herbal plants, the word “phytobiotics” is frequently used to describe the inherent bioactive compounds present in plants that have an impact on animal growth and health [2]. Herbal products are a common choice for dietary supplements in humans due to their non-toxic chemical makeup, low cost, and ease of accessibility. However, because phytogenics are a novel class of feed additives, we still do not fully understand how they function or how to include them in animal diets [3].

*Moringa oleifera *(MO), which belongs to the family *Moringaceae, *is commonly known as a horseradish tree or drumstick tree. It has both nutritional and medicinal values, with some useful minerals, vitamins, and amino acids. All segments of the MO tree, such as the fruit, leaves, seeds, bark, gum, leaf, and seed oil, have been utilized for the treatment of various diseases alone or in combination with other medicines in South Asia. This includes gastrointestinal, hematological, hepato-renal, and cardiovascular disorders, as well as the treatment of infectious, inflammatory, and inflammatory diseases [4,5]. It is capable of providing exceptional medicinal, industrial, and nutritional value for both human and animal food/feed consumption. It is rich in amino acids, beta-carotene, ascorbic acid, and vitamins. Because of its exceptional therapeutic and medicinal properties, it may be used as a medication to treat a variety of conditions. It can also be utilized as a growth promoter, antioxidant, and antimicrobial agent. The presence of caffeic acid and cinnamon acid gives it antioxidant benefits. It was reported that this plant has over 92 useful components, including 36 anti-inflammatory agents, 46 antioxidants, and 18 amino acids [6].

The MO leaf meal contains 86% dry matter, 22.4% crude fiber, 4.36% ether extract, 29.5% crude protein, 28.0% calcium, 0.23% phosphorus, and a very small amount of tannin, according to a study. It promotes immunity and has antibacterial properties. MO contains anti-inflammatory, anticancer, antiulcer, and antioxidant effects within its various extracts and powder forms [7]. MO leaf supplements in diets have indicated anti-inflammatory, antioxidant, anti-ulcerous, and anti-cancerous activities. Immune-modulatory and growth-promoting properties showed that it has no major side effects that endanger human life or livestock [8]. The dietary MO leaf powder supplemented in the feed or water of chickens has an improved effect on their production performance. Reducing the pathogen’s burden in the digestive tract also improves the villus height of the villus in the intestine tract (duodenum) and protects against different harmful microorganisms [9]. The MO leaf as a dietary supplement for broilers resulted in a notable enhancement in weight gain in comparison to the control (negative) group. The improvement can be linked to the elevated protein content found in MO leaves [10]. MO has several positive health factors, including the immune responses of the birds. Moreover, chick diet supplements with MO indicate greater performance (production) and immunity than untreated chicks [11]. The dietary leaf powder of MO might enhance physiological and physiochemical features as well as intestinal health in birds, which is linked to the Moringa leaf’s capacity to reduce inflammation in the intestinal tract [12].

MO leaf and seed extracts (SEs) have multiple characteristics due to their high fiber content, increased absorption, lower toxicity, and enhanced microflora of beneficial bacteria. The combination of MO seed and leaf extract is stable, and due to its bioavailability, it is expected to enhance the morphological parameters of the intestinal tract and gut microbiota. Therefore, this study is planned to find out the role of MO leaf and seed supplementation on growth traits, gut health, and microflora in broilers. As far as our knowledge is concerned, there has been no such research done on the use of MO seed and leaf extract in broilers.

## Materials and Methods

### Ethical approval

The Ethical Committee of the Department of Chemical and Life Sciences, Qurtuba University of Science and Information Technology, Peshawar, Pakistan, has approved this research trail. DR/393, dated April 12, 2022.

### Grouping and management of birds

This research study included 280 birds. The birds were weighed on arrival and randomly divided into seven (7) groups with eight (8) replicates in each group (*n =* 5). The humidity and temperature on day 1 were kept at 65% ± 5% and 35°C ± 1°C, respectively. The temperature dropped off by 3°C each week till it attained 26°C ± 1°C on day 21, with a humidity of 65% ± 5%, and stayed the same at the end of the trial.

### Preparation of M. oleifera leaf and SE

The MO leaves and stem was collected from Muzaffargarh, Punjab, Pakistan, and then taxonomically identified in the Department of Botany, Qurtuba University of Science and Information Technology, Peshawar. The leaf and seed were separately crushed to make powder. Subsequently, 500 gm of the powder was soaked in 5 l (5,000 ml) of ethanol for 48 hours and thoroughly mixed and filtered through a muslin cloth. The final extracted product was weighed and refrigerated for further use.

### Dietary strategy

Chicks were fed commercial basal diets (BDs) made from corn base; these diets were supplemented with varying concentrations of MO leaf extract and SE for 35 days. Group I was the control (untreated) group (fed merely BD), while Group II received 0.8% *M. oleifera *leaf extract (MLE); Group III was given 0.8% MSE; Group IV was given 1.2% MLE; Group V was given 1.2% MSE; Group VI was given 0.8% MLE + 0.8% MSE; and Group VII was given 1.2% MLE + 1.2% MSE.

### Parameters

The following parameters were examined: growth trait (body weight, body weight gain (BWG), feed intake, and feed conversion ratio), small intestine morphometry (height, width, and surface area of villus, crypt depth (CD), and height/CD ratio of villus, thickness of lamina propria (LP), muscularis mucosa, and externa), goblet cell counts in the small intestinal tract (acidic, mixed, and total goblet cell count), and gut microflora (*Escherichia coli*,* Lactobacillus*, and *Bifidobacteria*).

### Growth performance

The initial body weight was measured, and then it was measured every week for BWG. The feed utilization by birds was recorded daily. Recorded feed consumption and weight gained were used for the calculation of the feed conversion ratio (FCR).

FCR = Feed consumed/ BWG

### Sampling

On day 35, two birds from each replicate (16 birds/group) were selected for sampling. After cervical dislocation, the small intestine and ingesta were collected. From the duodenum (at the duodenal loop), jejunum (between duodenum and ilium), and ilium (distal segment before the ileocecal junction equaling the length of the cecum), about 2 cm portions were obtained and preserved in 10% buffered formalin for histological analysis as described by Ali et al. [13]. Ingesta from ilium was collected in normal saline and used for microbial counts.

### Preparation of intestinal sample slides for microscopic study

Tissue processing was performed using the paraffin embedding technique, according to Khan et al. [8]. All tissues were fixed in 4% paraformaldehyde in phosphate-buffered saline for about 24 hours and washed overnight in PBS. The tissue becomes dehydrated by immersion in its higher ethanol concentrations (60%, 70%, 90%, and 100%) each for 2 hours. The tissues underwent a clearing process in two rounds of xylene, each lasting 2 hours. Paraffin wax was employed for infiltration, and the tissues were immersed in molten paraffin using a hot air oven (58°C) for 6 hours. Plastic molds were utilized to create blocks, and subsequently, the tissue block was affixed to the microtome and rotated on the wheel. If the embedded intestinal tissue reached 20 μm, the block was adjusted to 5–6 μm to obtain a thin ribbon. A water bath (45°C) with a small amount of gelatin facilitated the transfer of the thin tissue ribbon after the microtomy. The resulting tissue section was mounted on a slide and appropriately labeled using a pencil. The slides were subjected to a hot air oven (90°C–100°C) for 15 minutes to achieve drying. The slides containing tissue sections were then stained through the hematoxylin and eosin (H&E) technique, following a protocol similar to that used by Khan et al. [7] with slight modifications.

### Morphometry of the intestinal tract

In each intestinal cross-section, five well-oriented villi were selected for measuring villus height (VH), width (VW), and CD. The VH (µm) was determined from the villus of the tip to the crypt junction of the villus. The CD (µm) was demarcated as the depth between nearby villi. From VH and CD, the VH/CD ratio was measured. Villus width (VW) was measured at three points, i.e., at the villus tip, at the midpoint of the villus, and at the base of the villus. An average of these three values was used as the width of the villus. The villus surface area (VSA) was determined by using the formula (2π) × (V/2) × (VL). The thickness of the LP and muscularis externa was measured [14]. For histo-morphometry, the slides were observed under a microscope with a 4X objective lens (Labomed USA), and for measurement of VH, VW, CD, and the thickness of LP, MM, and ME, using a commercial program (Labomed, PixelPro) software.

### Cellular differentiation in the intestinal tract (duodenal, jejunal, and ileal)

The slides were stained with combined Alcian blue-PAS for goblet cell counting and examined with a 10X objective lens. Acidic mucin-containing goblet cells (GC) are stained blue, neutral mucin-containing GC are stained magenta, and mixed GC with both neutral and acidic mucin stain purple [15].

### Gut microflora

The *Bifidobacteria *spp*.*,* Lactobacillus *spp., and* E. coli *counts were determined through different selected mediums. Using a traditional culturing procedure, digesta was taken from two birds per replicate for the counting of specified microbial diversity. The colony-forming unit formula was used to get the selected bacterial count [16].

### Statistical design

The data were analyzed using a one-way analysis of variance, and it was presented as mean SEM using Statistical Packages (SPSS Inc.) for Windows Version 13.3 (Chicago, IL, USA). Using significant differences (*p* < 0.05), the differences between the groups were measured.

## Results

### Growth traits

#### Body weights (BW)

In the first 3 weeks of the trial, body weights did not vary among the treated and control groups (Table 1). In the fourth week, body weight was increased (*p* < 0.05) in the D (BD + 1.2% MLE) and F (BD + 0.8% MLE + 0.8% MSE) groups than in the A (control) group. Moreover, the B (BD + 0.8% MLE) and E (BD + 1.2% MSE) groups showed greater (*p* < 0.05) BW than the A (control) group. In the last week (5^th^) of the trial, BW was increased (*p* < 0.05) in the F (BD + 0.8% MLE + 0.8% MSE) and B (BD + 0.8% MLE) groups than in the A (control) group.

#### Body weight gain

In the first two weeks, the BWG showed no significant changes among the control and treated groups (Table 2). In the third week, BWs were increased (*p* < 0.05) in the F (BD + 0.8% MLE + 0.8% MSE) group than in the A (control) and all treated groups. In the fourth week, the BWGs were higher (*p* < 0.05) in the B (BD + 0.8% MLE), D (BD + 1.2% MLE), E (BD + 1.2% MSE), and F (BD + 0.8% MLE + 0.8% MSE) groups than in the A (control) group. In the last week (5th), BWGs were also higher (*p* < 0.05) in the D (BD + 1.2% MLE) and F (BD + 0.8% MLE + 0.8% MSE) groups than in the A (control) group.

#### Feed intake

In the whole trial, feed intake did not vary among the control and treated groups (Table 3).

#### Feed conversion ratio

FCR between the treated and untreated (control) groups did not differ significantly during the first three weeks (Table 4). In the fourth week, FCR had improved (*p* < 0.05) among all treated groups compared to the A (control) and G (BD + 1.2% MLE + 1.2% MSE) groups. In the last week (5th), FCR was improved (*p* < 0.05) in the B (BD + 0.8% MLE), D (BD + 1.2% MLE), and F (BD + 0.8% MLE + 0.8% MSE) groups than in the A (control) group.

### Gut morphometry

#### Duodenum

In the duodenum, VW, muscularis mucosa, thickness of the externa, and CD did not vary among the treated and control groups (Table 5). The D (BD + 1.2% MLE) and F (BD + 0.8% MLE + 0.8% MSE) groups have higher VH (*p* < 0.05) than the A (control) group. Furthermore, VH was also higher (*p* < 0.05) in B (BD + 0.8% MLE), and C (BD + 0.8% MSE) groups than in the A (control) group. Duodenal villus, surface area, and ratio of VH/CD were higher (*p* < 0.05) in B (BD + 0.8% MLE), D (BD + 1.2% MLE), and F (BD + 0.8% MLE + 0.8% MSE) groups than in the A (control) group. LP thickness (LPT) was also higher (*p* < 0.05) in B (BD + 0.8% MLE), C (BD + 0.8% MSE), D (BD + 1.2% MLE), and F (BD + 0.8% MLE + 0.8% MSE) groups than in the A (control) group.

#### Jejunum

In the Jejunum, VW, surface area, thickness of muscularis mucosa, externa, and LP remained unaffected in the experiment among the treated and control groups (Table 5). VH, surface area, and ratio of VH/CD were higher (*p* < 0.05) in the D (BD + 1.2% MLE) and F (BD + 0.8% MLE + 0.8% MSE) groups than in the A (control) group. The jejunal CD was increased (*p* < 0.05) in A (control) and G (BD + 1.2% MLE + 1.2% MSE) groups than in all other treated groups.

**Table 1.** Effect of leaf and seed powder extracts of *M. oleifera* on body weight (grams) of broiler chickens.

Week	A	B	C	D	E	F	G	SEM	*p*-value
First	137.40	139.60	142.20	136.33	138.50	142.33	136.60	1.2	0.119
Second	382.32	400.61	392.28	406.16	388.20	398.83	380.39	2.8	0.385
Third	726.50	746.50	719.38	756.30	738.66	775.63	726.22	4.8	0.188
Fourth	1180.25^c^	1292.20^b^	1245.30^bc^	1310.38^ab^	1296.33^b^	1344.83^a^	1198.38^c^	9.5	0.020
Fifth	1650.60^d^	1767.20^c^	1717.30^d^	1808.60^b^	1768.38^c^	1864.30^a^	1668.60^d^	11.3	0.002

^a–d^ Means with different superscripts within the same row are significantly different (*p* < 0.05) from each other.

A = Basal Diet (BD), B = BD + 0.8% *M. oleifera* leaf extract (MLE), C = BD + 0.8% *M. oleifera* seed extract (MSE), D = BD + 1.2% MLE, E = BD + 1.2% MSE, F = BD + 0.8% MLE + 0.8% MSE, G = BD + 1.2% MLE + 1.2% MSE.

**Table 2.** Effect of leaf and seed powder extracts of *M. oleifera* on BWG (grams) of broiler chickens.

Week	A	B	C	D	E	F	G	SEM	*p*-value
First	90.40	91.38	92.20	85.30	86.33	92.40	89.60	1.0	0.186
Second	244.92	261.01	250.08	269.83	249.70	256.50	243.78	1.8	0.194
Third	344.18^ b^	345.89^ b^	327.10^ b^	350.14^b^	350.46^ b^	376.80^ a^	345.84^b^	2.5	0.028
Fourth	461.75^c^	545.70^ab^	525.92^b^	554.08^a^	557.67^a^	569.20^a^	472.16^c^	6.8	0.015
Fifth	462.35^b^	475^b^.00	472^b^.00	498.22^ab^	472.05^b^	519.47^a^	470.22^b^	7.7	0.003

^a–c^Means with different superscripts within the same row are significantly different (*p* < 0.05) from each other.

#### Ileum

In the ileum, significant differences were not observed in the thickness of the LP, muscularis mucosa, externa, VSA, and CD among the treated and control groups (Table 5). In B (BD + 0.8% MLE), D (BD + 1.2% MLE), and F (BD + 0.8% MLE + 0.8% MSE) groups, the VH was increased (*p* < 0.05) than in the A (control) group. VW was increased (*p* < 0.05) in A (control) and G (BD + 1.2% MLE + 1.2% MSE) groups than in the other all treated groups. Ileal VH/CD was higher (*p* < 0.05) in the B (BD + 0.8% MLE) and F (BD + 0.8% MLE + 0.8% MSE) groups than in the A (control) group.

#### GC count in the intestinal tract

Duodenal, jejunal, and ileal mixed goblet (MG) cell counts did not vary among the treated and control groups (Table 6). Duodenal and jejunal acidic goblet cell (AG) and total goblet cell (TG) numbers were increased (*p* < 0.05) in B (BD + 0.8% MLE), D (BD + 1.2% MLE), and F (BD + 0.8% MLE + 0.8% MSE) groups than in the A (control) group. Ileal AG and TG cell numbers were increased (*p* < 0.05) in B (BD + 0.8% MLE), C (BD + 0.8% MSE), D (BD + 1.2% MLE), and F (BD + 0.8% MLE + 0.8% MSE) groups than in the A (control) group.

#### Bacterial cell count

The count of *Bifidobacter* did not differ (*p* < 0.05) among the treated and untreated (control) groups (Table 7). The *Lactobacillus *counts were higher (*p* < 0.05) in the B (BD + 0.8% MLE), E (BD + 1.2% MSE), and F (BD + 0.8% MLE + 0.8% MSE) groups than the A (control) group. *E. coli *counts were decreased (*p* < 0.05) in all treated groups compared to the A (control) and G (BD + 1.2% MLE + 1.2% MSE) groups. However, group G (BD + 1.2% MLE + 1.2% MSE) also had lower (*p* < 0.05) *E. coli *counts than the A (control) group.

## Discussion

The study aims to investigate the effect of dietary MO on the growth traits and gut health of the chickens. In the current experiment, in the last of the fourth and fifth weeks, body weight was increased (*p* < 0.05) in Groups D (BD + 1.2% MLE) and F (BD + 0.8% MLE + 0.8% MSE) than in the A (control) group. In the current study, in the starter phase of the trial, body weight did not vary among the supplement groups. These results are endorsed by Ochi et al. [10], who documented that the significant decrease in chick weight in the starter phase (first 3 weeks of the trial) did not vary among the treated groups. This may be because MO contains tannins and phytates, which are considered antinutritional factors. These factors decreased the absorption of nutrients in the birds at an early age. The current study is similar to Agashe et al. [4], who claimed that the body weight of the birds was increased due to the dietary leaf of MO. This is due to the fact the fact that moringa has significant amounts of vitamins, iron, proteins, and minerals.

In the current study of the first two weeks, the BWG did not vary among the control and treated groups. In the last three weeks of the experiment, BWGs were also higher (*p* < 0.05) in the groups D (BD + 1.2% MLE) and F (BD + 0.8% MLE + 0.8% MSE) than the A (control) group. Similar results were also mentioned by Khan et al. [8], who observed that MO did not affect BWG in the starter phase. This may be due to the early age of the birds; the gut is not fully developed. Another study showed that MO supplements improved the weight gain of the chicks. This is caused by the active ingredients that play an important role in the improvement of digestion, metabolism, and nutrient absorption through the gut [1]. Another study showed that BWG did not vary with dietary supplements of MSE during the growing phase. This effect is due to the high dose of MO and the improper processing of the leaves, which increased the levels of anti-nutritional factors that resulted in the reduction of utilization and digestion of feed [17].

**Table 3.** Effect of leaf and seed powder extracts of *M. oleifera* on feed intake of broiler chickens.

Week	A	B	C	D	E	F	G	SEM	*p*-value
1^st^	126.83	122.83	122.33	118.50	118.14	124.50	121.70	0.9	0.280
2^nd^	364.33	360.16	361.50	352.60	357.16	354.00	363.30	1.2	0.255
3^rd^	636.30	620.60	607.20	591.80	625.30	589.36	630	3.9	0.182
4^th^	847.80	808.36	795.30	790.53	822.50	810.40	850.60	7.5	0.100
5^th^	1075.80	896.60	990.80	965.20	1008.30	939.70	1090.50	9.5	0.212

**Table 4.** Effect of leaf and seed powder extracts of *M. oleifera* on FCR of broiler chickens.

Week	A	B	C	D	E	F	G	SEM	*p*-value
1^st^	1.40	1.34	1.33	1.38	1.37	1.35	1.36	0.01	0.830
2^nd^	1.49	1.38	1.45	1.31	1.43	1.38	1.49	0.01	0.080
3^rd^	1.85	1.79	1.85	1.69	1.78	1.56	1.82	0.01	0.060
4^th^	1.84^b^	1.48^a^	1.51^a^	1.43^a^	1.48^a^	1.42^a^	1.80^b^	0.02	0.040
5^th^	2.33^c^	1.90^ab^	2.10^b^	1.94^ab^	2.14^bc^	1.82^a^	2.32^c^	0.03	0.020

^a–c^Means with different superscripts within the same row are significantly different (*p* < 0.05) from each other.

**Table 5.** Effect of leaf and seed powder extracts of *M. oleifera* on morphometric parameters of intestine in broiler chickens.

Parameters	A	B	C	D	E	F	G	SEM	*p*-Value
Duodenum									
VH (µm)	911.60^ d^	1218.80^ bc^	1188.50^ c^	1269.80^ ab^	1174.20^ c^	1330.60^ a^	902.50^ d^	17.5	0.002
VW (µm)	117.10	147.30	135.40	141.60	133.50	138.80	118.10	2.3	0.320
VSA (mm)^2^	335.90^c^	564.80^a^	505.90^b^	565.60^a^	492.90^b^	580.10^a^	335.70^c^	14.60	0.003
CD (µm)	215.60	170.50	191.30	177.40	194.80	167.60	216.30	3.9	0.110
LPT (µm)	82.21^b^	89.20^ab^	92.10^a^	91.70^a^	85.30^b^	96.10^a^	81.10^b^	1.9	0.045
MMT (µm)	37.70	28.30	30.43	29.60	31.35	28.60	28.50	0.9	0.120
MET (µm)	162.58	142.16	148.33	150.41	139.91	141.83	162.58	1.9	0.200
VH:CD	4.22^c^	7.15^a^	6.21^b^	7.15^a^	6.03^b^	7.93^a^	4.17^c^	0.3	0.001
Jejunum									
VH (µm)	758.57^ c^	920.20^b^	900.56^b^	983.30^a^	848.23^bc^	998.85^a^	757.45^c^	19.4	0.030
VW (µm)	134.50	127.20	131.41	128.55	128.58	127.33	133.50	3.5	0.225
VSA (mm)^2^	320.90^b^	368.20^ab^	372.70^a^	397.10^a^	342.40^ab^	399.40^a^	318.5^b^	11.40	0.049
CD (µm)	143.50^ a^	111.10^bc^	121.60^bc^	109.85^bc^	132.80^ab^	103.60^c^	141.40^a^	2.0	0.019
LPT (µm)	76.30	90.50	102.70	81.40	87.60	92.45	78.20	1.7	0.135
MMT (µm)	36.40	26.10	32.06	25.65	30.48	27.40	35.60	0.9	0.088
MET (µm)	165.35	134.80	157.30	138.22	161.35	131.20	163.25	2.0	0.07
VH:CD (µm)	5.29^c^	8.28^b^	7.41^b^	8.95^a^	6.39^bc^	9.64^a^	5.36^ c^	0.4	0.019
Ileum									
VH (µm)	565.70^c^	866.20^a^	739.30^b^	795.50^ab^	708.15^b^	813.40^ab^	559.20^c^	18.3	0.002
VW (µm)	138.20^a^	120.52^bc^	121.30^bc^	127.20^b^	116.55^c^	125.00^b^	139.40^a^	2.0	0.009
VSA (mm)^2^	245.50	328.50	282.10	318.10	259.50	319.50	245.80	6.25	0.135
CD (µm)	134.10	110.70	128.00	114.85	110.60	109.70	130.60	1.8	0.080
LPT (µm)	64.50	78.75	69.10	71.80	72.75	83.90	65.00	1.5	0.350
MMT (µm)	39.90	30.50	36.20	30.40	35.80	28.70	38.50	0.6	0.090
MET (µm)	180.30	154.70	166.70	160.60	162.50	153.00	175.00	1.9	0.088
VH:CD	4.21^c^	7.82^a^	5.78^b^	6.93^ab^	6.40^ab^	7.41^a^	4.30^c^	0.3	0.010

^a–c^Means with different superscripts within the same row are significantly different (*p* < 0.05) from each other.

VH(Villus height); VW(Villus width); VSA(Villus surface Area); CD(Crypt Depth); LPT(Lamina propria thickness); MMT(Muscularis mucosa thickness); MET(Muscularis Externa thickness); VH:CD(Villus height:crypt depth ratio).

In the trial, feed intake did not vary among the control and treated groups. Ullah et al. [6], reported the same finding, they concluded that MLE reduced feed intake in the whole trial. Feed intake is reduced due to the use of a high concentration of MO in the diet of broilers, which might have harmful effects due to a weakened taste when included in the feed of broilers. Another study reported that feed intakes were not significant when the birds were treated with different levels of MO. Moringa supplements have shown no toxic results or contain some factors that prevent the intake of feed in opposition to the absorption of nutrients [8,18].

**Table 6.** Effect of leaf and seed powder extracts of *M. oleifera* on GC containing mixed and acidic mucin in small intestine of broilers.

Intestinal segment	Goblet cell	A	B	C	D	E	F	G	SEM	*p*-value
Duodenum	AGC	61.10^b^	97.20^a^	90.20^ab^	99.30^a^	88.30^ab^	105.50^a^	65.00^b^	2.9	0.010
	MGC	35.90	49.20	43.10	47.50	39.20	51.90	36.60	1.2	0.050
	TGC	97.00^ c^	146.40^a^	133.30^ab^	146.90^a^	127.50^b^	156.40^a^	101.60^c^	3.2	0.020
Jejunum	AGC	82.20^c^	137.60^a^	127.50^ab^	139.30^a^	112.80^b^	146.30^a^	85.60^c^	4.3	0.020
	MGC	49.80	52.30	48.00	43.50	46.40	40.60	49.90	1.2	0.240
	TGC	132.00	189.90	175.50	182.80	159.20	186.90	135.50	4.3	0.080
Ileum	AGC	90.90^c^	155.40^ab^	147.85^ab^	176.10^a^	124.20^b^	170.10^a^	95.30^c^	5.0	0.013
	MGC	47.30	51.50	39.60	47.20	40.80	51.20	51.50	1.0	0.190
	TGC	138.20^c^	206.90^a^	187.45^ab^	223.30^a^	165.00^b^	221.30^a^	146.80^c^	5.9	0.012

^a–c^ Means with different superscripts within the same row are significantly different (*p* < 0.05) from each other.

**Table 7.** Effect of leaf and seed powder extracts of *M. oleifera* on bacterial cell counts of broiler chickens.

*Bacterial population*	A	B	C	D	E	F	G	SEM	*p*-value
*Lactobacilus*	6.85^b^	8.09^a^	7.50^ab^	7.80^ab^	7.38^b^	8.06^a^	7.00^b^	0.05	0.025
*Bifidobacteri*a	7.32	7.78	7.46	7.57	7.66	7.72	7.45	0.07	0.590
*Escherichia coli*	8.05^c^	7.25^a^	7.62^ ab^	7.48^ab^	7.49^ab^	7.27^a^	7.89^b^	0.04	0.042

^a–c^Means with different superscripts within the same row are significantly different (*p* < 0.05) from each other.

In the current experiment last week, FCR was improved (*p* < 0.05) in B (BD + 0.8% MLE), D (BD + 1.2% MLE), and F (BD + 0.8% MLE + 0.8% MSE) groups than in the A (control) group. A similar study also reported that moringa supplementation in the diet of chickens improves production performance. Improvement in feed efficiency and weight gain in the feed of broilers by having MO supplements, which improve the FCR in broilers [19]. In another similar study, Eladia et al. [11], also reported that *Moringa* leaf meal showed better FCR as compared to the control broiler diet. Moringa leaf meal contains natural growth promoters, immune and beneficial bacteria-stimulant activities that enhance growth performance.

The current research revealed that, in the duodenal segment, the villus, surface area, and ratio of VH/CD were higher (*p* < 0.05) in B (BD + 0.8% MLE), D (BD + 1.2% MLE), and F (BD + 0.8% MLE + 0.8% MSE) groups than in the A (control) group. A similar study documented that gut histomorphometry in broilers is vital for the utilization of nutrients and absorption and designates the good physiology of the birds. Higher villi are valuable for better nutrient absorption because they enhance the surface area and improve the intestinal health of broilers [7]. In the duodenum, the VSA and the ratio of VH/CD are improved due to the MLE supplementation in the feed of broilers in comparison to birds whose diet contains no MO leaf powder or extract [20]. The higher ratio of VH/CD made the intestinal architecture more oriented toward digestion and absorption and improved the potential of hydrolysis, thus requiring fewer nutrients for gut maintenance [21].

In the jejunum, the VH, surface area, and the ratio of VH/CD were higher (*p* < 0.05) in D (BD + 1.2% MLE) and F (BD + 0.8% MLE + 0.8% MSE) groups than in the A (control) group. Kavoi et al. [22], endorsed our finding and stated that the *Moringa* supplementation in the diets of the birds increased the surface area of the villus, which is freely available for the absorption of nutrients, and the ratio of VH/CD is a key factor in the gut functional capability, and a decrease in the ratio is considered harmful to the nutrient’s digestion and absorption in the chickens. The mucosal intestinal wall, whose capability to produce movement locally and cause the mucosal folding to enhance the contact between the luminal content and the wall of the epithelium to enhance absorption [23].

In the current study, in groups B (BD + 0.8% MLE), D (BD + 1.2% MLE), and F (BD + 0.8% MLE + 0.8% MSE), the VH of the ileum was increased (*p* < 0.05) than in the A (control) group. A similar study documented that dietary *Moringa *leaf increased the length and width of the villus (duodenal and ileal) in broilers. The intestinal tract absorbs the nutrients through digestion with the fragments of feed in the duodenum, fermentation, and consequently the absorption in the ileum of the fermented products [24]. The MLE contains glutathione contents, a conjugate component of glutamate, the richest amino acid in the circulation of the blood, which plays an important role in maintaining intestinal mucosal integrity [25].

The ability of MO to enhance growth traits, successfully prevent intestinal morphological atrophy, relieve oxidative damage, and downregulate mRNA expression of mucosal inflammatory genes in the jejunum under oxidative stress [12]. The findings indicate that there were notable variations in growth characteristics and feed costs. Additionally, it demonstrates that the leaf meal of *moringa *may easily replace up to 15% of the costlier protein sources in the diet without sacrificing performance [26].

The duodenal and ileal tissue, AG, and TG numbers were increased (*p* < 0.05) in B (BD + 0.8% MLE), D (BD + 1.2% MLE), and F (BD + 0.8% MLE + 0.8% MSE) groups. The current study was endorsed by Zeeshan et al. [27], who found out that the GC in the mucosa of the intestine indicates the capacity of the intestine to produce mucin. GCare is an important element of the intestinal immune system. Mucin (glycoprotein) is produced on the superficial layers of the intestinal mucosa of the broilers. It plays an important role in lubricating the intestinal mucosa and identifying and inhibiting the colonization of pathogenic bacteria. Acidic GC can protect against harmful bacteria by showing resistance to the destructive action of the bacteria’s proteases. Another study showed that MO leaf supplementation enhanced the counts of total GC. This improvement may be due to the presence of vitamins in MO, which play an important role in the development and maturation of mucin cells, as documented by Khan et al. [8]. The inclusion of MSE in the broiler’s diet promoted mucin production, which protects the lumens of the intestinal tracts. The higher mature GC (Acidic), containing acidic mucin due to the dietary supplementation of *moringa* seed, contributed to the reduction in the pH of the intestine, which may lead to an increase in the uptake of nutrients and enhance the microbial profile in the intestine [21].

The *Lactobacillus *counts were higher (*p* < 0.05) in the B (BD + 0.8% MLE), E (BD + 1.2% MSE), and F (BD + 0.8% MLE + 0.8% MSE) groups than the A (control) group. *Escherichia coli *counts were decreased (*p* < 0.05) in all treated groups compared to the A (control) and G (BD + 1.2% MLE + 1.2% MSE) groups. A similar study reported that MLE and MSE have antibacterial activity. The supplementation of MSF and MSE significantly increased the number of beneficial bacteria (*Lactobacillus, Bifidobacteria*) and decreased the number of pathogenic bacteria (*Salmonella *spp*., E. coli*) [20]. MO is rich in fiber, which is a substrate for beneficial bacteria. When the beneficial bacterial population is increased, it causes the expulsion of harmful bacteria, and the beneficial bacteria also cause the production of volatile fatty acids [8]. The volatile fatty acids acted on enterocytes and increased the surface area of the villus, resulting in increased absorption, which led to growth and enhanced the immune status of the birds [1,30]. MO enhances the activity of antioxidants such as catalase and superoxide dismutase. These antioxidant enzymes have effects on free radicals, viruses, and pathogenic bacteria in broilers [19]. The count of *Lactobacillus *was higher in the group supplemented with MLE, and counts of *E. coli *and Salmonella spp. were decreased as compared to the control. The herbal supplements prevent pathogenic bacteria colonization, develop the outer barrier, and inhibit the nutrient supply for the growth of bacteria [20,28].

Metagenomics provides a new perception of microbiomes and the interactions between microbes and their hosts. Dietary nutrition, such as phytobiotics (*M. oleifera*), has a positive influence on the composition of the intestinal microbiota, which is important for gut health maintenance and improving the immune status of the host. The beneficial microbiota such as *Lactobacillus spp., Bacteroides fragilis*, *Butyricoccus pullicaecorum,* and *Bacteroides barnesiae *had higher populations than the pathogenic ones such as *Clostridium* and *Salmonella spp. *with phytobiotic supplements like MO*, *it is important to modulate the immune system, protect the gut health from pathogenic microbes, and improve the gut health by increasing the population of beneficial bacteria, which leads to an increase in the production of volatile fatty acids that cause villus hypertrophy and improve the nutrients [29,30].

## Conclusions

The leaf extract of MO group D (MLE–1.2% in the diet of the broilers) and the combined use of MLE and MSE, group F (BD + 0.8% MLE + 0.8% MSE) is superior for enhancing the broilers performance, the architecture of the intestine, and mucosal protection of the gut. The MO seed and leaf extract reduced the load of pathogenic bacteria in the intestinal tract and improved the beneficial gut *microflora* due to the higher fiber content of this medicinal plant. It is also a great stress-reducing agent and is used as an antibiotic replacement.

## List of Abbreviations

BD, Basal diet; CD, Crypt Depth; LPT, Lamina propria thickness; MMT, Muscularis mucosa thickness; MET, Muscularis Externa thickness; MLE, *Moringa oleifera *leaf extract; SE, Seed extract; VH, Villus height; VW, Villus width; VSA, Villus surface Area; VH: CD, Villus height: Crypt depth ratio.
